# Millimetric scale two-photon Bessel-Gauss beam light sheet microscopy with three-axis isotropic resolution using an axicon lens

**DOI:** 10.1117/1.NPh.10.3.035002

**Published:** 2023-06-23

**Authors:** Cléophace Akitegetse, Thomas Charland, Mireille Quémener, Charles Gora, Véronique Rioux, Michel Piché, Yves De Koninck, Martin Lévesque, Daniel C. Côté

**Affiliations:** a Centre de recherche CERVO, Québec, Québec, Canada; b Centre d’Optique, Photonique et Laser, Québec, Québec, Canada; c Zilia inc., Québec, Québec, Canada; d Université Laval, Département de Physique, Génie Physique et d’Optique, Québec, Québec, Canada; e Université Laval, Département de Psychiatrie et Neurosciences, Québec, Québec, Canada

**Keywords:** light sheet, axicon, two-photon, Bessel-Gauss

## Abstract

**Significance:**

Typical light sheet microscopes suffer from artifacts related to the geometry of the light sheet. One main inconvenience is the non-uniform thickness of the light sheet obtained with a Gaussian laser beam.

**Aim:**

We developed a two-photon light sheet microscope that takes advantage of a thin and long Bessel-Gauss beam illumination to increase the sheet extent without compromising the resolution.

**Approach:**

We use an axicon lens placed directly at the output of an amplified femtosecond laser to produce a long Bessel-Gauss beam on the sample. We studied the dopaminergic system and its projections in a whole cleared mouse brain.

**Results:**

Our light sheet microscope allows an isotropic resolution of 2.4  μm in all three axes of the scanned volume while keeping a millimetric-sized field of view, and a fast acquisition rate of up to $34\;{\mathrm{mm}}^{2}/s$. With slight modifications to the optical setup, the sheet extent can be increased to 6 mm.

**Conclusion:**

The proposed system’s sheet extent and resolution surpass currently available systems, enabling the fast imaging of large specimens.

## 1 Introduction

In the last two decades, interest in light sheet microscopy has remarkably increased in neuroscience due to its rapid and high-resolution imaging capabilities of complex biological samples.^1^^–^^12^ The main feature of a light sheet microscope is the selective illumination of a thin plane in the sample. The configuration of this type of microscope consists of two perpendicular arms, one for the illumination and one for the detection. The illumination arm forms a sheet of light in the sample, whereas the detection arm collects the emitted signal and forms the image of the illuminated plane on the camera. In general, the thickness of the sheet is much smaller than the depth of field of the detection objective, resulting in an axial resolution dependent on the light sheet thickness.

Although there has been tremendous technological advances in recent years, few light sheet systems are able to capture information from very large fields of view (FOV) of several millimeters while featuring a cellular resolution. Indeed, to meet these requirements, the sample must be illuminated with a long and thin beam, which is incompatible with the use of diffractive beams, such as Gaussian beams. After describing historical developments in light sheet microscopy, we will describe a light sheet system that overcomes the limitations of previous designs and achieves a very large FOV with cellular resolution.

The first description of a light sheet microscope dates back to 1902, when Siedentopf and Zsigmondy observed gold particles using sunlight projected through a slit.^13^ In 1964, McLachlan observed bismuth dendrites by forming a line of light at the focal plane of an imaging lens, through a slit, and scanning the sample to create a virtual light sheet and to form an image.^14^

In 1993, Voie and his colleagues used a light sheet microscope to study the internal structures of the cochlea of gerbils,^15^ which had been cleared with a five-part solution of methyl salicylate and of three parts of benzyl benzoate. This microscope consisted of a collimated laser beam illuminating the sample through a cylindrical lens. With this system, they succeeded to obtain a wide FOV of 750  μm with, for the time, an impressive axial resolution of 26.1  μm.

Huisken et al. described a system they called selective plane illumination microscopy, which also uses a cylindrical lens to illuminate the sample perpendicularly to the axis of the imaging objective. With this system, they imaged the heartbeat and green fluorescent protein (GFP)-labeled ganglion cells of live medaka fish embryos as well as the embryogenesis of a *Drosophila melanogaster* embryo over a 17-hr period. With a thickness between 3 and 10  μm, the light sheet had an extended 60-μm FOV, that enabled three-dimensional reconstructions of samples up to a cubic millimeter. Dodt et al. published in 2007 another version of a light sheet microscope.^16^ They used a cylindrical lens to form the sheet in the sample and, to reduce the shadow artifacts created by the single-sided illumination, they added another illumination arm on the opposite side. They used their microscope in combination with benzyl alcohol/ benzyl benzoate (BABB) clearing to detect unique GFP-tagged neurons in excised mouse hippocampi. The width and thickness of the sheet varied depending on the size of the sample. For large samples like mouse brains, the thickness was set between 25 and 50  μm, whereas for smaller samples, the thickness could be as low as 6  μm. The same year, Huisken and Stainier found another solution to the shadow artifact problem by rotating the cylindrical lens around the sample and combining different views of the sample.^17^

However, the use of a cylindrical lens for light sheet formation requires a slit in front of the lens to form the illumination profile. This has the effect of blocking the laser power, resulting in a low illumination efficiency of the order of 3%. To overcome this issue, Keller and his colleagues published in 2008, a study where they recorded the location and movement of nuclei in whole zebrafish embryos during the first 24 hrs of development using another type of light sheet microscope.^18^ In their version, a Gaussian beam focused on the sample by a low numerical aperture objective was scanned at high speed to create a virtual sheet. This configuration allowed for a highly increased illumination efficiency of 95%. Still, using a Gaussian illumination, the system has been adapted in several ways to increase the FOV by illuminating the sample bidirectionally^19^ and using a mixed-wavelength excitation to achieve multi-color two-photon fluorescence.^4^

In all strategies described above to produce a light sheet, the thickness of the sheet corresponding to the waist size of a focused Gaussian beam will limit the length over which this size can be maintained (i.e., the depth of field, here the Rayleigh range of the focused beam). This will ultimately limit the axial resolution of the microscope. A compromise is usually necessary between the minimum thickness of the light sheet and its uniform illumination field. To remedy this problem, the thickness of the light sheet could be decreased and, by scanning the sample in-plane, the resulting mosaic of small images could be reconstructed to obtain a larger image.^20^ This strategy allows one to image volumes of several cubic millimeters with an axial resolution of 2  μm, although at a very slow speed. For instance, it was reported to take 50 s to image a section of 0.96×1.88  mm, making it unpractical to image a larger biological sample such as an entire mouse brain. A tunable focal length lens to scan the excitation sheet in the direction of its propagation has also been used to create a larger virtual light sheet.^21^ Rather than trying to enlarge the virtual sheet, Gao and his colleagues used a spatial light modulator to move a small sheet in the direction of the light propagation and to create image mosaics.^8^ This strategy is also employed by Voigt et al. in their mesoSPIM system in which an electrically tunable lens extends the FOV to 13.29 mm at the expense of a larger sheet thickness of 6.52  μm.^22^

In 2010, the self-reconstruction properties of Bessel-Gauss beams in dense non-homogeneous media, were reported. The scanned beam improved the image quality and penetration in high scattering media.^12^^,^^23^ Few years later, isotropic 3D images were generated using a scanning Bessel-Gauss beams light sheet microscope.^24^ By combining Bessel-Gauss beams with structured illumination and two-photon fluorescence, they were able to generate a sheet less than 0.5  μm thick and were able to image the dynamics of small intracellular structures including mitochondria, filopodia, vesicles, and mitotic chromosomes with an isotropic resolution of 300 nm. Interest in this microscope was, however, in its capacity to reach high isotropic resolution in small volumes with FOVs as small as 60×80  μm. There was therefore no demonstration of the applicability of this method to image a larger volume, such as an entire mouse brain.

A better contrast was reported by combining a Bessel-Gauss beam with two-photon fluorescence than one generated with a Gaussian beam.^25^ By using this approach, a 246  μm wide sheet and 2.8  μm sheet thickness were obtained; the FOV was large enough to image the pharyngeal muscle of *Caenorhabditis elegans*. However, this method used an average laser power two orders of magnitude larger than the conventional selective plane illumination microscope using a Gaussian beam, which can provoke photobleaching and phototoxicity.

Using an axicon lens, a rotationally symmetric prism producing a focal line along its optical axis, a recent study reported a line length of 600 to 1000  μm while maintaining an axial resolution 2−3  μm.^26^ Another aspect to consider for an optimal light sheet imaging system is the acquisition time required to perform volumetric imaging. Considering the acquisition time to be determined by the sheet’s extent of $1\;{\mathrm{mm}}^{2}$ and the exposure time chosen between 100 to 300 ms, we calculated that the system was able to obtain surface rates between 3.3 and $10\;{\mathrm{mm}}^{2}/s$. This method of calculating the acquisition rate, not attributing the delay due to the sample movement, will be used throughout the paper to better compare the systems.

The Airy beam, which is propagation invariant like the Bessel-Gauss beam, was also used in a light sheet system to achieve a FOV of ∼200  μm with a sheet thickness of 1.5  μm.^7^ By imaging actin fibers and nucleic acid in a juvenile amphioxus, Vettenburg showed that the asymmetric illumination pattern of the airy beam contributed to a better image contrast. In another recent study, using non-orthogonal objectives with Gaussian beams and a virtual light sheet, a sub-micrometer lateral resolution could be generated with an axial resolution slightly under 3  μm.^27^ Such high resolution, however, comes at the cost of a smaller FOV of less than $500\;\mu m^{2}$ of length. Thus far, none of the previous light sheet systems developed were able to combine the generation of large FOV images with a small axial resolution.

Here, we show that using an amplified femtosecond laser to take advantage of the decoupling of the diameter and the length of the Bessel-Gauss beam, we were able to obtain up to 5.845 millimeter-sized FOV, a 2.4-μm axial resolution, and a fast acquisition rate of up to $34\;{\mathrm{mm}}^{2}/s$, surpassing currently available systems.

## 2 Theory

### 2.1 Generating a Millimeter-Long Thin Bessel-Gauss Beam Using an Axicon

**Fig. 1:**
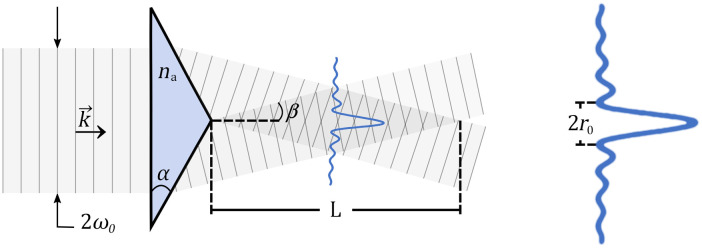
Generation of a Bessel beam of focusing angle β by an axicon of refractive index $n_{a}$ and base angle α using an incident plane wave of half-width $\omega_{{}^{\circ}}$. The beam radial intensity profile is represented vertically as a Bessel function and is maintained over the length of the darker grey region with a central lobe of width $2r_{0}$.

At the core of our light sheet microscope is an amplified laser (RegA Coherent, 250 kHz, 4  μJ per pulse) that goes through an axicon to generate a long, thin line of light that is scanned to obtain a sheet. Any ray refracted by the axicon is deflected toward its axis of revolution, by an angle β characteristic of the axicon. The interference of the multiple plane waves forming a cone around the optical axis generate a Bessel beam (Fig. 1). In practice, a collimated Gaussian beam of radius $\omega_{{}^{\circ}}$, is projected onto an axicon, thus creating a Bessel-Gauss beam.

The focusing angle β of the generated Bessel-Gauss beam can be derived with simple geometry and is expressed as $\arcsin(n_{a}\;\sin\;\alpha )-\alpha$.

To calculate the main characteristics of the Bessel-Gauss beam, such as its axial length L and its central lobe width $2r_{0}$, we first use the analytical expression in cylindrical coordinates of the intensity distribution at the output of the axicon^28^

$$I(r,z)=2\pi k(\sin^{2}\;\beta )zI_{{}^{\circ}}\;\exp(-\frac{2z^{2}\;\sin^{2}\;\beta }{\omega_{{}^{\circ}}^{2}})J_{0}^{2}({\mathrm{krn}}_{a}\;\sin\;\beta ),$$ (1)

where k=2π/λ. The half-width of the central lobe is obtained from the first zero of the Bessel function of the first kind of order zero and is $r_{{}^{\circ}}=2.41\lambda /(2\pi n_{a}\;\sin\;\beta )$. By setting the cylindrical coordinate r to zero, one gets the on-axis intensity profile

$$I(z)=2\pi k(\sin\;\beta {)}^{2}zI_{{}^{\circ}}\;\exp(-\frac{2(\sin^{2}\;\beta )z^{2}}{\omega_{{}^{\circ}}^{2}}).$$ (2)

The full-width at half maximum (FWHM) length L of the generated Bessel-Gauss beam for two-photon excitation is $0.58\times \omega_{{}^{\circ}}/\tan\;\beta$.^29^ One observes that the Bessel-Gauss beam central lobe size ($r_{{}^{\circ}}$) is decoupled from its axial length L. Both depend on the focusing angle β but $r_{{}^{\circ}}$ is independent of $\omega_{{}^{\circ}}$, the incident Gaussian beam half-width. It is therefore possible, with the same axicon, to modify the length of the generated Bessel-Gauss beam by adjusting the incident beam width, without impacting the width of the generated beam central lobe.

However, to generate a Bessel-Gauss beam with an effective length L of 1 mm and a central lobe half-width $r_{{}^{\circ}}$ of 1  μm with a 800-nm laser, the necessary combination of incident beam half-width and axicon base angle would be impractical.^29^ Indeed, the incident Gaussian beam would be so narrow that only the tip of the axicon would be illuminated and the cone angle would be near 90 deg. Such an acute angle is difficult to manufacture because the sharp axicon tip often breaks, resulting in a rounded tip. The narrow beam is thus mostly refracted by the imperfect tip, creating a poor-quality Bessel-Gauss beam, which more closely resembles the focal point of a Gaussian beam. A previous study showed that it was possible to use an axicon coupled to a Fourier transform lens inserted into a laser scanning microscope to create an extended depth of field.^29^ The position of the axicon-lens pair must be carefully set at the entrance of the optical system to exploit the magnification factors of the telescopes in the microscope that scale down the length of the focal line. This allows the use of an axicon with a lower base angle in combination with a larger input beam, without compromising the core radius of the Bessel-Gauss beam.

**Fig. 2:**
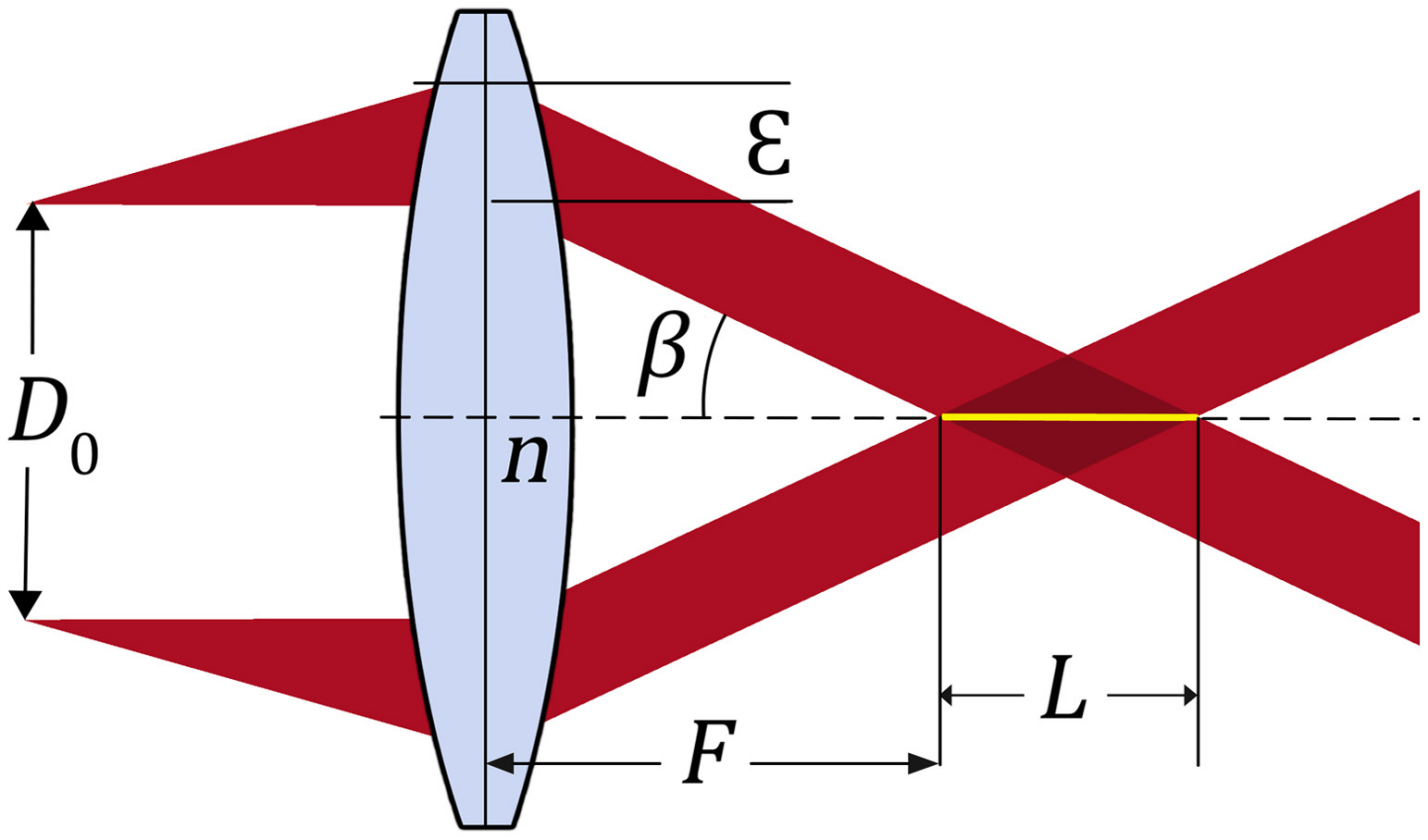
Focusing an annular beam of inner diameter $D_{{}^{\circ}}$ and of thickness ε with an angle β using a lens of focal length F to create a Bessel-Gauss beam of effective length L.

Here, we exploit the magnification factor of our system to generate a long and thin focal line. An axicon that has a base angle α and a refractive index $n_{a}$ conjugated to a Fourier transform lens of focal length $f_{\alpha }$ projects an annular beam with a diameter $D_{g}=2f_{\alpha }\;\tan\;\beta$. This annular beam is then magnified with an angular magnification M and projected to the back aperture of an objective. The annular beam at the back aperture of the objective has a thickness ε and inner diameter $D_{O}$. The objective forms a Bessel-Gauss beam by focusing the ring (Fig. 2) in a medium of refractive index n. The beam has characteristics of a Bessel-Gauss beam over a total distance of $\varepsilon /{\mathrm{NA}}_{B}$, where ε is the thickness of the annular beam at the back aperture of the lens and ${\mathrm{NA}}_{B}=n\;\sin(\beta )$.

When considering the beam core radius and length requirements, we can determine the dimensions $D_{O}$ and ε of the annular beam at the back aperture of the lens. Consequently, when considering a lens or an objective, it is important to calculate the corresponding $D_{O}$ parameter and to ensure that the annular beam fits in the rear aperture.

For an optimal design, five parameters must be carefully chosen. They are: the incident Gaussian beam half-width on the axicon ($\omega_{{}^{\circ}}$), the base angle of the axicon (α), the focal length of the Fourier-Transform lens ($f_{\alpha }$), the angular magnification between the axicon-Fourier lens and the back aperture of the objective (M), and finally, the focal length of the objective (F).

## 3 Methods

### 3.1 Light Sheet Microscope Using an Axicon Lens

The laser source is a regenerative amplifier (Coherent Inc. RegA 9000) seeded by a titanium: sapphire laser (Coherent Inc. Mira 900-F), itself pumped by a ${\mathrm{Nd}:\mathrm{YVO}}_{4}$ laser (Coherent Inc. Verdi V18). The output of the RegA (800 nm, 4  μJ, <200  fs, 250 kHz), widened by a Galilean telescope, is incident on the axicon. The axicon is the starting point for the design of the optical system. We chose an axicon with a base angle of 2.5 deg (Edmund Optics) and refractive index of 1.4533, which falls into manufacturers typical specification, ranging from 1 deg to 5 deg. A 200 mm Fourier transform lens paired with the axicon form a ring on a galvanometric mirror (Thorlabs GVS011). Having β=0.0198, it is possible to calculate that a Fourier transform lens with a focal length of 200 mm will project the beam into a ring with a diameter $D_{0}$ of ∼8  mm. The ring is relayed to the rear aperture of the excitation objective (Olympus XLFLUOR 4×/340, 0.28 NA) through a 2.78× magnification Keplerian telescope, consisting of a pair of lenses (54 and 150 mm). After magnification, the ring has a diameter of 22 mm, which matches the 25-mm rear aperture of the excitation objective. The latter focuses the ring to form a Bessel-Gauss beam into the sample. The laser power at the sample was 200 mW, which corresponds to a pusle energy of 0.8  μJ. By scanning the galvanometric mirror, a light sheet is formed. A second objective (Olympus XLPLN10XSVMP 0.6 NA) is placed perpendicular to the first objective to detect the fluorescence emitted from the illuminated plane. A tube lens (Olympus U-TLU) focuses the detected fluorescence signal on a scientific CMOS camera (Hamamatsu ORCA-Flash4.0 V2). To help the design of the light sheet microscope, one can use the Raytracing Python module.^30^ It allows to test various axicon base angles, to easily determine the required focal length of the Fourier transform lens, and obtain a light ring with a diameter that matches the rear aperture of a given objective.

The Bessel-Gauss beam is scanned using a GSV-011 galvanometric system (Thorlabs Inc.) with a scanning angle of up to ±20  deg at a frequency of 130 Hz. The position of the scanning mirror is controlled by an electrical signal (0.5 V per degree) generated by an Inputs/Outputs device (PCIe-6351, National Instruments). The waveform of the output signal is controlled by a homemade control and acquisition software.

A MPC-385 micromanipulation system (Sutter Instruments) is used for positioning. This includes a MP-285 micromanipulator, a MPC-200 controller as well as a ROE-200 module for manual control and communication with a computer. Communication with a computer is achieved through a RS-232 serial port emulated via a USB link.

The cleared tissues are in a homogenizing liquid at all times, even during the imaging process. We designed a 3D-printed acrylonitrile butadiene styrene imaging chamber that keeps the sample immersed in the liquid. The imaging chamber has a parallelepipedal shape with three openings: two openings on two perpendicular faces for positioning the objectives and an opening on the top for filling and positioning the sample. The latter is mounted in a quartz glass cuvette filled with homogenization liquid. The cuvette is held by a holder fixed on the 3D micromanipulator. This allows the cuvette with the sample to be moved by the micromanipulation system without having to move the objectives and the imaging chamber.

For the ultra large FOV experiments, we modified the system described above as follows:

• The Fourier transform 200-mm lens is replaced with a 125-mm lens. This allows a 5.845-mm-long Bessel-Gauss beam in the sample.
• The imaging chamber is removed from the setup so that the cuvette containing the cleared sample is not submerged in the homogenizing liquid. The detection lens used is an air objective.
• The 10× multi-immersion detection objective is replaced by a 2× objective (Olympus MVPLAPO2XC 0.5 NA) and the 180-mm tube lens is replaced by a 45 mm one to create a 4× magnification.
• A 0.63× coupler is used between the tube lens and the camera, which decreases the effective magnification to 1.26×.

### 3.2 Cleared Mouse Brains

The mouse brains were dissected on postnatal day 1, then treated as described in the iDISCO protocol.^31^ Briefly, after brain extraction and fixation in 4% paraformaldehyde, a methanol pretreatment was performed before immunostaining. Then, the permeabilization and the blocking were carried out before an incubation period of 7 days in primary rabbit anti- tyrosine hydroxylase (TH) antibodies (Pel-Freez P40101, 1:250). After one day of washing in PTwH buffer solution (100 ml of 10X PBS, 2 ml of Tween20, 1 ml of 10  mg/ml heparin stock solution), the brains were incubated in an anti-rabbit secondary antibody coupled to the Alexa Fluor 594 (Invitrogen A-21207, 1:400) for 6 days. After washing in PTwH, the brains were transferred to a clarifying solution for imaging.

## 4 Results

### 4.1 Large FOV Isotropic Two-Photon Light Sheet Microscopy

**Fig. 3:**
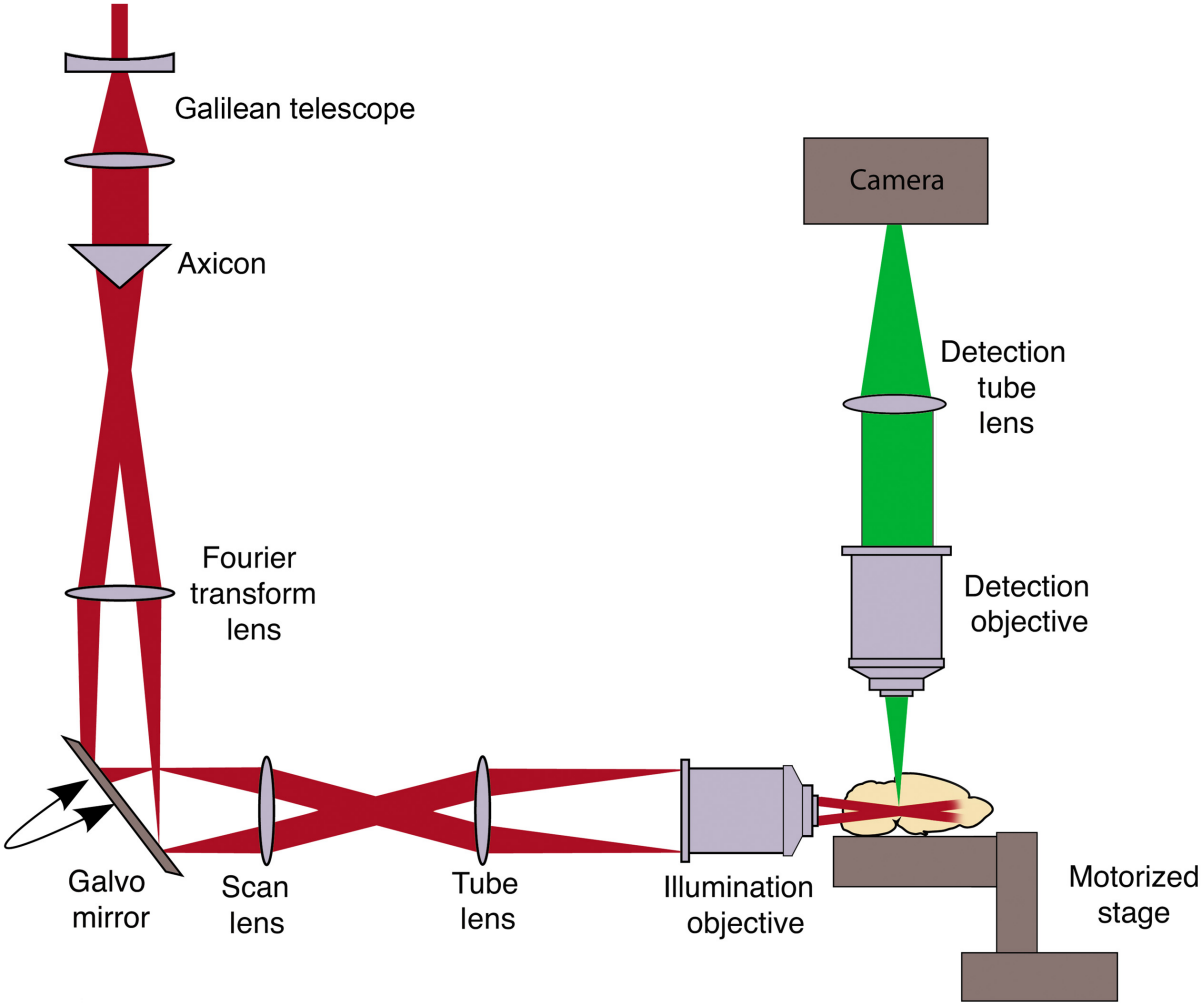
Schematic representation of our light sheet setup: a Gaussian beam is expanded by a Galilean telescope and then refracted by an axicon into a Fourier transform lens, which forms a ring on a galvanometric mirror. The ring is passed through the scan lens and the tube lens, which constitute a Keplerian telescope that magnifies the annular beam. It is then projected at the back aperture of an objective that focuses it as a Bessel-Gauss beam into the sample. A light sheet can be created by moving the galvanometric mirror to scan across the sample plane. This first section is the illumination arm. The second section is the collection arm, where the fluorescence emitted by the sample is gathered by an objective and is focused back into a camera to acquire an image. A motorized stage allows to adjust the position of the sample in relation to the focal distances of both objectives.

**Fig. 4:**
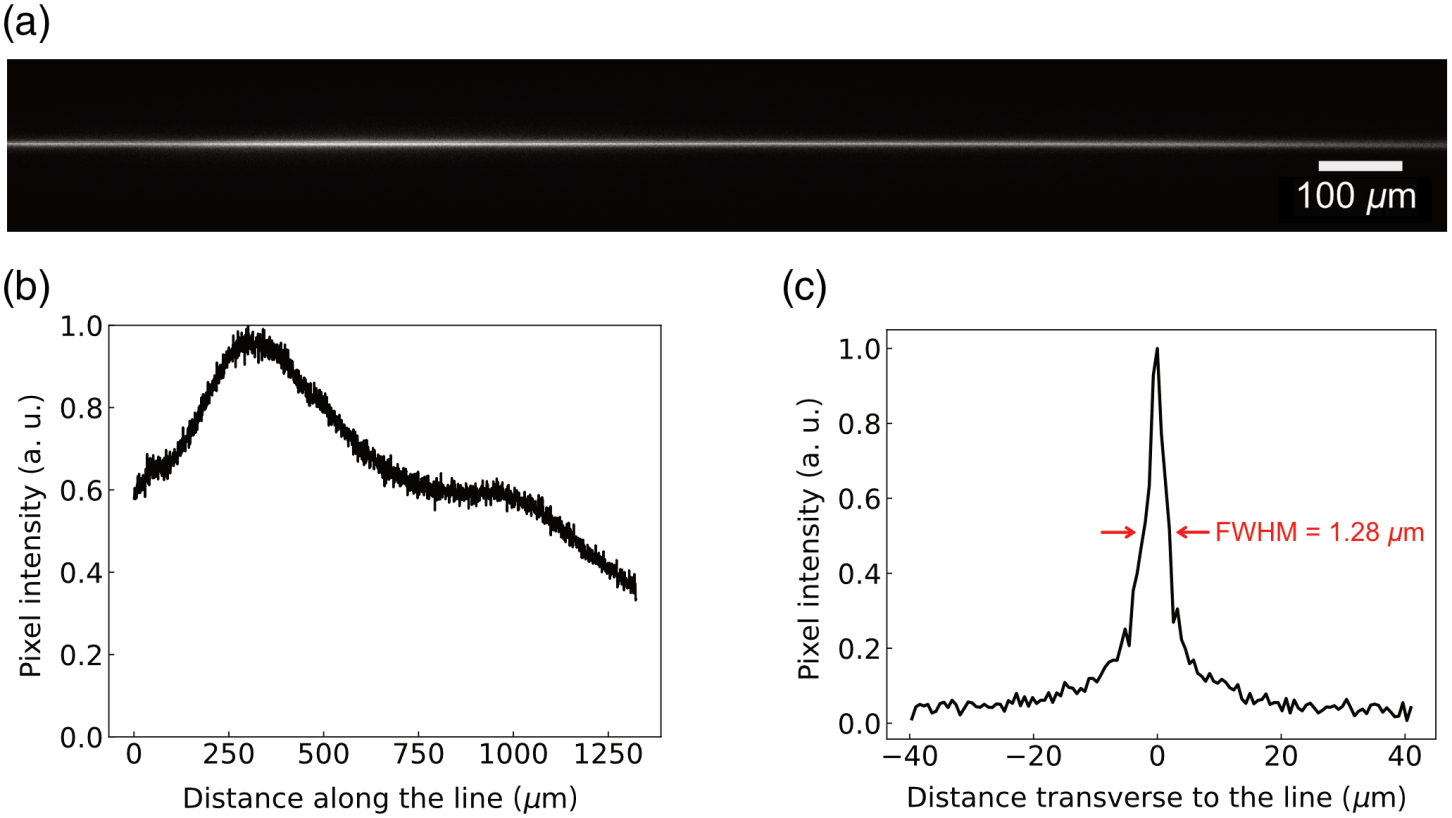
(a) Focal line of the large FOV light sheet microscope featuring a 1.35 mm long beam imaged by the camera. Note that the line extends slightly beyond the FOV of the camera. (b) Longitudinal profile of the Bessel-Gauss beam. (c) Transverse profile of the Bessel-Gauss beam. The FWHM of approximately 1.28  μm is considered to be the sheet thickness when scanning the beam.

We designed a large FOV light sheet microscope (Fig. 3) by choosing optics to form, into the sample, a 1.35 mm long beam of 1.28  μm radius to achieve the largest possible FOV with the best resolution considering the available off-the-shelf optics. The focal line can be visualized in Fig. 4. This is achieved by using an excitation objective of focal length F=45  mm, an angular magnification M=0.36, a Fourier-Transform lens of focal length $f_{\alpha }=200\;\mathrm{mm}$, an axicon with base angle α=2.5  deg, and an incident Gaussian beam of half-width $\omega_{{}^{\circ}}=2.5\;\mathrm{mm}$. To get enough power to generate two-photon fluorescence on such a long line, we used an amplified femtosecond pulse laser source emitting at 800 nm.

The beam that exits the amplified laser is first widened by a Galilean telescope and is then refracted by the axicon. The Fourier-transform lens conjugated to the axicon shapes the beam into an annular shape on a galvanometric mirror. The annular shape is relayed to the back aperture of a long working distance excitation objective through a Keplerian telescope (angular magnification M). The excitation objective focuses the annular shape to form a Bessel-Gauss beam into the sample. By scanning the galvanometric mirror, the long and thin needle of light forms a light sheet in the sample. A 10× objective is placed perpendicular to the first objective to detect the fluorescence emitted from the illuminated plane. A 180 mm tube lens focuses the detected fluorescence signal on a scientific complementary metal–oxide–semiconductor (sCMOS) camera. In this setup, the FOV is limited by the camera. The latter indeed has 2048 pixels by 2048 pixels, each pixel being 6.5  μm by 6.5  μm. This is equivalent to 1.312 by 1.312 mm on the sample when applying the 10× magnification factor.

**Fig. 5:**
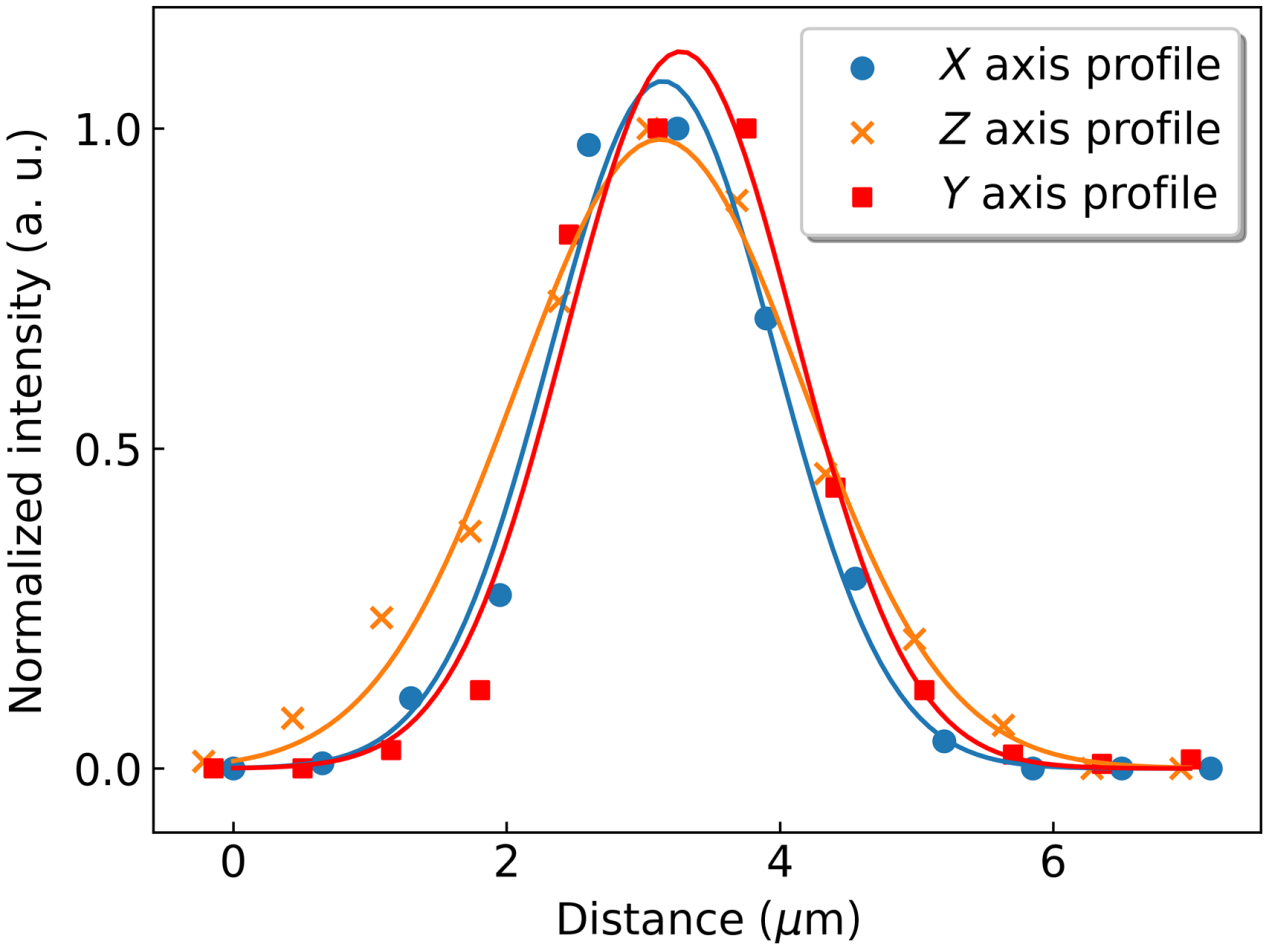
Resolution assessment using the average profiles of 500 nm beads measured along the three axis of a volume acquired with a 0.5  μm z-step. The sample consisted of a 1% w/w agarose gel with modified carboxylate yellow-green fluorescent microspheres. The solid lines correspond to Gaussian fits on the data.

The spatial resolution was evaluated by measuring the point spread function of the optical system using fluorescent beads. A phantom sample was prepared using a 1% w/w agarose gel (Invitrogen) containing 500 nm modified carboxylate yellow-green fluorescent microspheres (Invitrogen). We acquired a stack of many images with a step of 0.5  μm along the Z axis, from which the lateral and axial profiles of 90 beads were obtained. Each profile was fitted to a Gaussian curve, where the FWHMs were calculated and averaged (Fig. 5). A FWHM of (1.9±0.4)  μm was obtained laterally (XY), whereas it was (2.4±0.3)  μm axially (XZ).

**Fig. 6:**
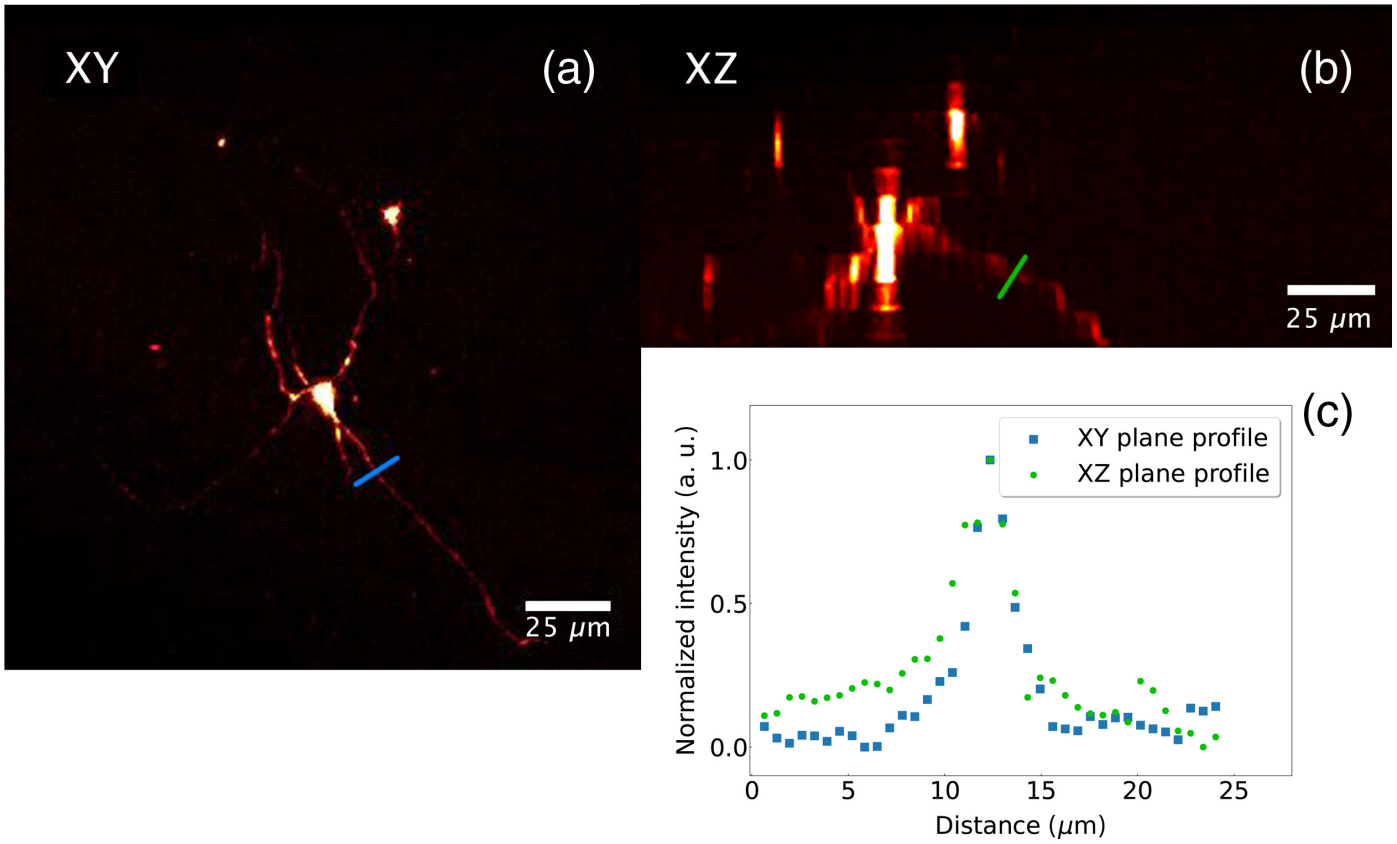
Neuron in a mouse brain slice injected with biocytin and imaged by the Bessel-Gauss beam scanning microscope. (a) Maximum intensity projection in the XY plane. (b) Maximum intensity projection in the XZ plane. (c) Intensity profile along the colored lines passing over the cell extensions in both the XY (blue) and the XZ (red) planes.

Neurons are particularly challenging cells to image due to their dendrites and axon that extend over several millimeters. It is thus of particular interest to be able to image the entirety of a neuron from the cell body to the dendrites and the axon. To test the ability of our light sheet system to resolve, in three dimensions, micrometric-scale structures, we injected the neuronal tracer biocytin on living 1-mm brain slices using whole cell patch clamp.^32^ The slice was then fixed, mounted in an agarose gel, cleared using the iDISCO protocol and imaged using our microscope. As can be seen in Fig. 6(a), a clear image of a neuron including its dendrites and part of its axon can be obtained within a thick brain section. By plotting the profile of a line perpendicular to one of the extensions of the cell in XY and XZ projection planes [Fig. 6(b)], the measured dimensions are 2.75  μm in the XY plane and 3.20  μm in the XZ plane. This result indicates that our system can discriminate small cellular profiles such as dendrites and axons.

By plotting the profile of a line perpendicular to one of the extensions of the cell in the XY and XZ projection planes [(Fig. 6(b)], the measured dimensions are 2.75  μm in the XY plane and 3.20  μm in the XZ plane. This confirms the system’s ability to resolve, in 3 dimensions, micrometric-sized structures.

**Fig. 7:**
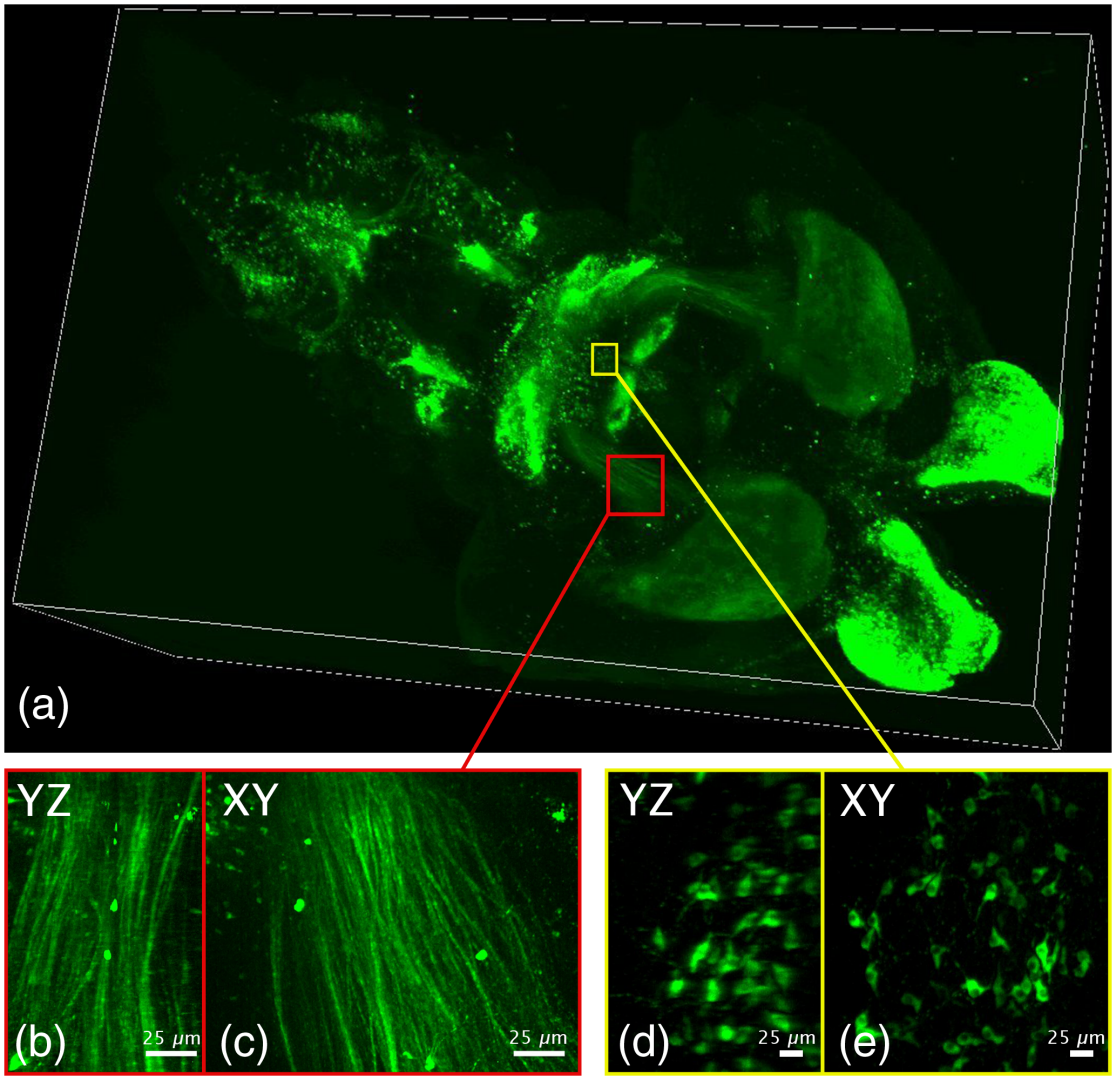
High-resolution isotropic imaging of the cleared brain. Data of a volume of 5.00  mm×5.00  mm×4.50  mm were acquired from a newborn cleared mouse brain at a magnification of 10×. The brain was cleared using the iDISCO protocol and stained with anti-TH antibodies coupled to Alexa-594. All the images were acquired in approximately 3 hours. (a) 3D view of the entire volume. (b-c) Maximum projection intensity of the volume in yellow box in the YZ (b) and XY (c) planes. (d-e) Maximum projection intensity of the volume in red box in the YZ (d) and XY (e) planes. The camera exposure time was set to 100 ms; ethyl cinnamate (refractive index n = 1.558) was used as the immersion medium.

**Fig. 8:**
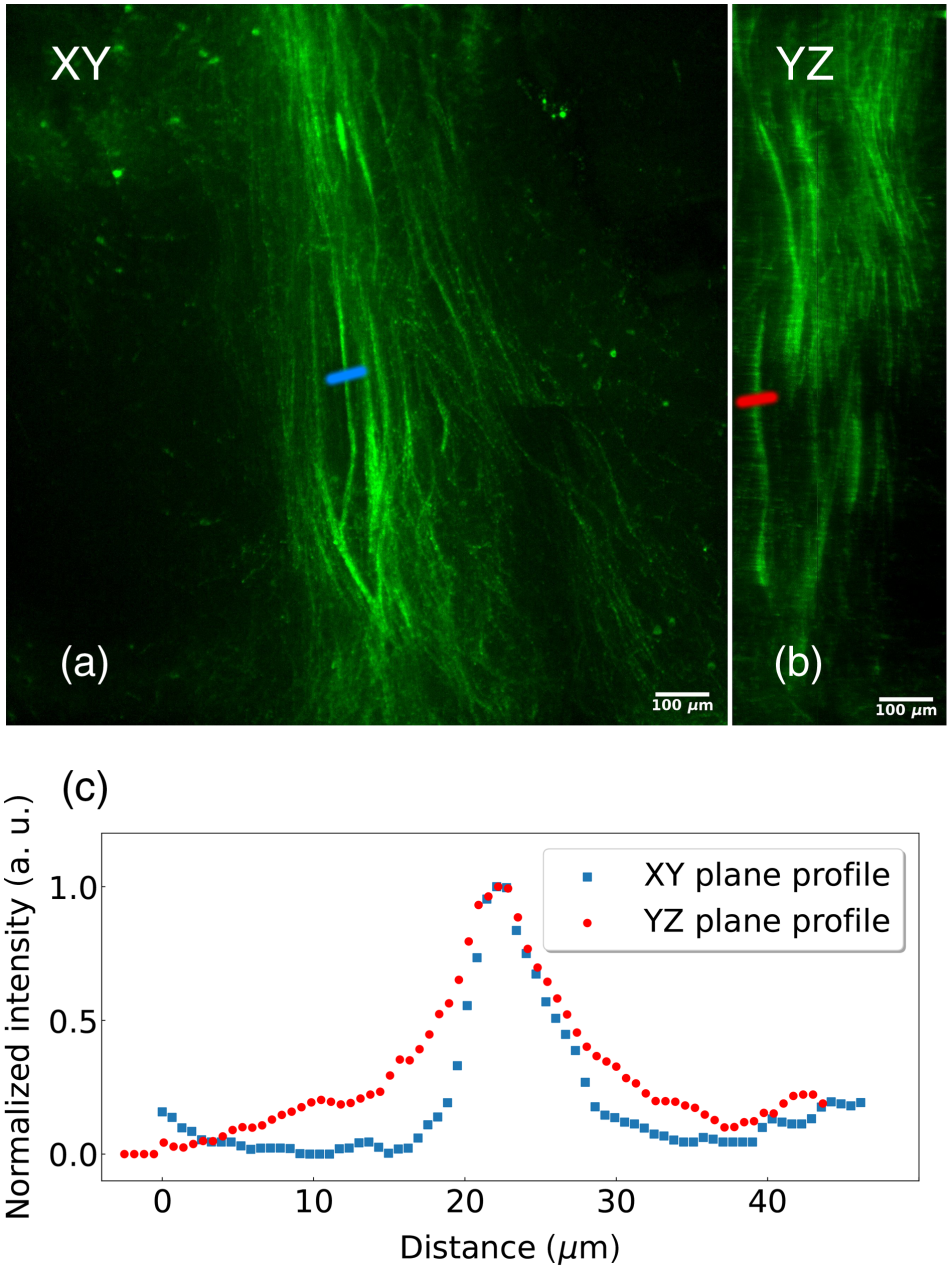
Dopaminergic axon bundles from Fig. 7. Maximum intensity projection of a 20  μm thick virtual section of the (a) XY and (b) YZ planes. (c) Intensity profile along the colored lines inserted over the fibers in the XY (blue) and YZ (red) planes.

The microscope was then used to acquire a volume of 5.00  mm×5.00  mm×4.50  mm from an intact, cleared newborn mouse brain. Using the iDISCO method,^31^ an antibody against tyrosine hydroxylase (TH) was used to label dopaminergic neurons and their projections in the brain. Sixteen images (4×4), acquired with an exposure time of 200 ms, were necessary to reconstruct an entire horizontal plane of the brain. With a step of 1  μm, 4,500 planes were acquired, for a total of 72,000 images. This resulted in just over 500 gigabytes of data acquired in ∼3 hours. As can be seen in Fig. 7, the high isotropic resolution is maintained. The axon bundles [Figs. 7(b) and 7(c)] as well as the cell bodies [Figs. 7(d) and 7(e)] are shown in the maximum intensity projection in the XY and YZ planes. There is no noticeable difference of resolution between the two perpendicular planes. To ensure this, we choose one of the dopaminergic axon bundles in the image and plotted the profile along a line perpendicular to the axon bundles in the XY and YZ planes (Fig. 8). According to these profiles, small axon bundles have a width of 7  μm in the XY plane and 9  μm in the YZ plane, confirming again the isotropic resolution of our microscope (Fig. 8).

**Fig. 9:**
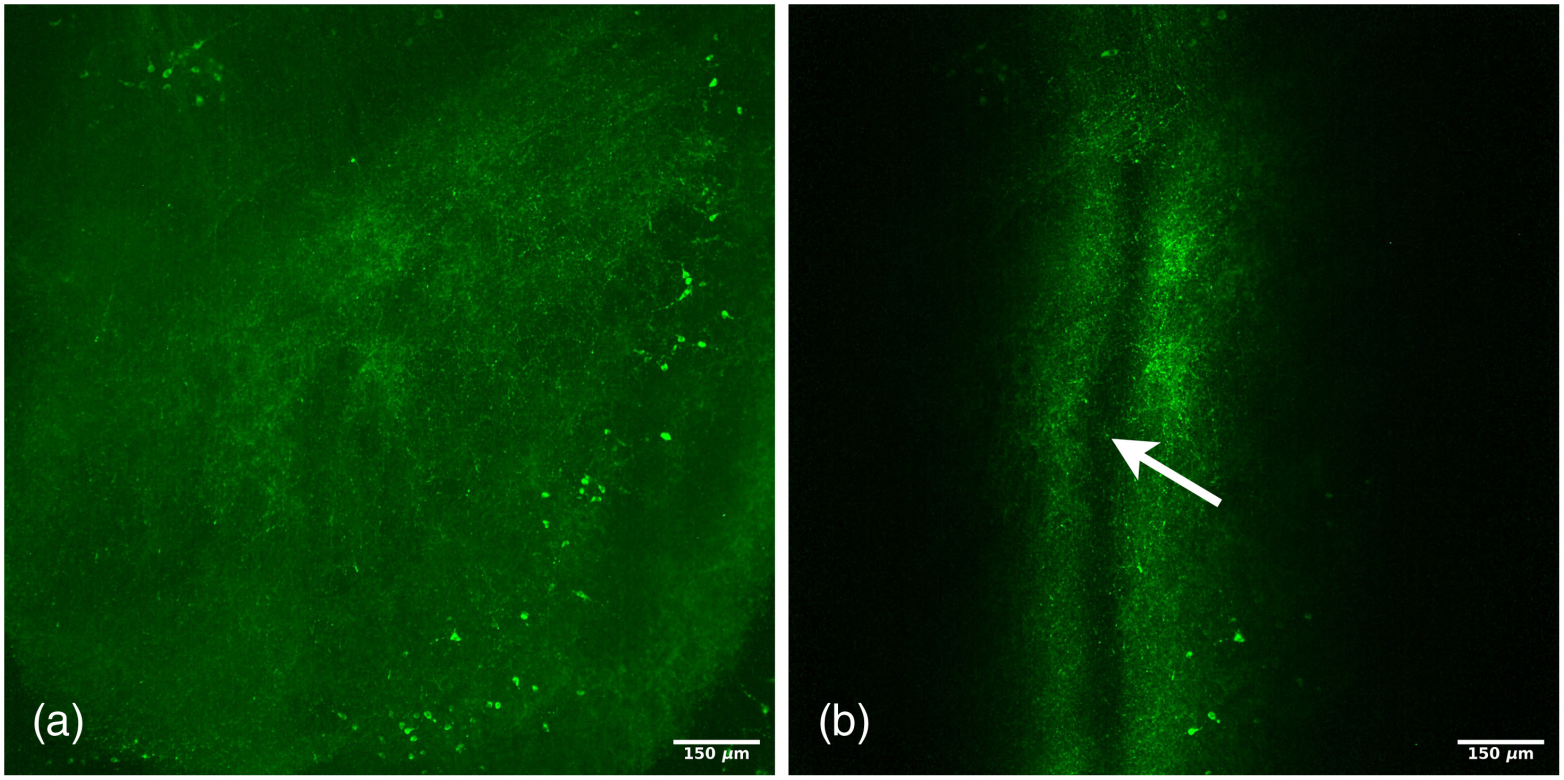
Comparison of two-photon fluorescence generated by a Bessel-Gauss beam and by a Gaussian beam. The sample was a postnatal day 1 mouse brain stained with anti-TH antibodies according to the iDISCO protocol. (a) Maximum intensity projection of a section imaged by scanning vertically a horizontal Bessel-Gauss beam. (b) Maximum intensity projection of the same section imaged instead by scanning a Gaussian beam of the same average power. The illuminated section in (b) is narrower since the generated line length is shorter. As indicated by the white arrow, the effect of photobleaching can be seen in the darker vertical line at the center of the image where the energy is more concentrated.

We have chosen two-photon microscopy to benefit from its reduced photobleaching and scattering in tissues. Moreover, the two-photon excitation is much weaker in the secondary lobes of the Bessel beam compared to the single-photon excitation, as discussed previously.^33^ In our case, the side lobes of the Bessel-Gauss beam were not intense enough compared to the main lobe to produce noticeable fluorescence. Additionally, the high power of the laser also allows to obtain a large depth of illumination by distributing the energy over the focal line of a Bessel-Gauss beam. To highlight the benefits of using such an illumination over traditional Gaussian beam excitation, we removed the axicon and acquired new images. Both techniques are compared in Fig. 9. When the axicon is removed, it is necessary to decrease the illumination power because the very high energy of the Gaussian beam can damage the sample. The power required to generate a fluorescence signal over only a quarter of the FOV is already sufficient to damage tissue at the waist of the beam [Fig. 9(b)]. With the Bessel-Gauss beam configuration, no photobleaching was observed.

### 4.2 Modified Setup: Ultra Large FOV Two-Photon Light Sheet Microscopy

Given the decoupling between the length and the diameter of a Bessel-Gauss beam, it is theoretically possible to lengthen the line of the setup described above, by modifying only the width of the incident beam on the axicon. For instance, to get a 1 cm long beam, enough to illuminate an entire section of an adult mouse brain, the half-width of the incident Gaussian beam on the axicon should be 20 mm. Optics used in the current setup, however, only have a radius of 12.5 mm at most. To demonstrate experimentally that it is possible to form such long lines, we have chosen to modify our setup by replacing the Fourier-transform lens. While decreasing the focal length, the latter reduces the required width of the incident Gaussian beam to a more practical value without having to change the diameter of the optical components. Thus, the Fourier transform lens has a focal length of 125 mm and the half-width of the Gaussian beam $\omega_{{}^{\circ}}$ is 4.5 mm. To be able to see the whole illuminated section, the detection objective as well as the tube lens were replaced to get an effective magnification of 1.26×.

**Fig. 10:**
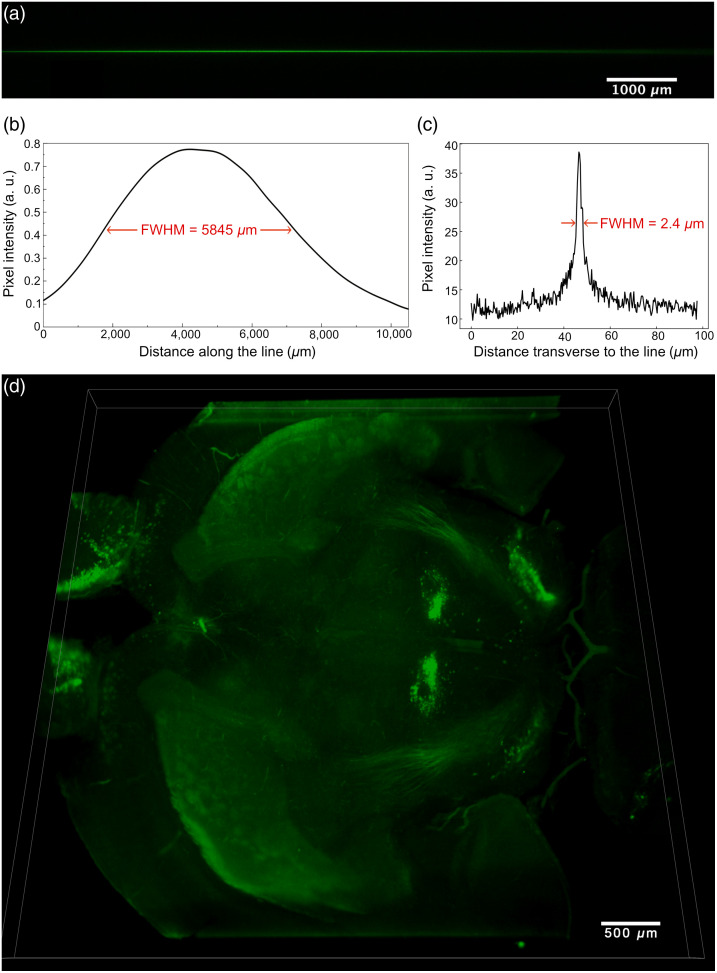
Very large FOV of a 5.28  mm×5.28  mm×0.268  mm volume data acquired from an intact clarified postnatal day 1 mouse brain with 2.52× magnification. The incident laser beam is expanded to 12 mm so that (a) if not scanned, a long thin (2  μm thick) Bessel-Gauss beam is imaged by the camera. (b) Longitudinal profile of the Bessel-Gauss beam. The FWHM of 5.845 mm is considered to be the effective length of the beam and thus the sheet’s extent. (c) Transverse profile of the Bessel-Gauss beam. The FWHM of ∼2.4  μm is considered to be the sheet’s thickness when scanning the beam. (d) Image of the olfactory bulb to the mid-brain of the intact mouse brain, without any tiling. (Video 1) Camera exposure time was set to 1 s. (Video 1, MP4, 10.9 MB [URL: https://doi.org/10.1117/1.NPh.10.3.035002.s1]).

To characterize this modified setup, a fluorescent solution was prepared in a cuvette and placed in front of the objectives. A long, thin line is formed in the fluorescent solution and is imaged by the camera. We get a usable line that is over a centimeter long with an FWHM a little less than 6 mm [Fig. 10(a)].

The detection tube lens is then modified to get a magnification factor that matches the FWHM of the illumination Bessel-Gauss beam. This allows us to align the detection FOV with the most intense portion of the excitation beam to reduce intensity non-uniformity in the images, for an effective magnification of 2.52×. As can be seen in Figs. 10(b) and 10(c), the generated sheet has an extent of 5.845 mm while the thickness is still approximately of 2.4  μm. Such a large sheet allows one to obtain a complete view of an entire adult mouse brain section with a mosaic of only two images by two images.

We then used this modified setup to obtain three-dimensional images of an entire mouse brain. Mouse brains were prepared, cleared, immunostained with a TH antibody, and revealed with a secondary antibody coupled to Alexa Fluor 594. With this configuration, we were able to image a horizontal region comprising the two striatum and from the olfactory bulb to the midbrain in a single image [Fig. 10(d)]. In a single FOV, we were able to observe simultaneously the cell bodies of dopaminergic neurons of the midbrain and their axons toward the forebrain in both hemispheres while maintaining an optical sectioning of 2.4  μm. This represents a major advance over pre-existing techniques that, to maintain good sectioning, must limit the FOV to a few hundred microns.

Moreover, this large FOV increases greatly the acquisition speed that can reach more than $34\;{\mathrm{mm}}^{2}/s$ while considering a 5.845-mm sheet length and a longer one second exposure time. However, these impressive characteristics come at the expense of a lower lateral resolution, which is now greatly limited by the camera. It has a pixel size of 6.5  μm, which corresponds to a pixel size of 2.58  μm on the sample. Thus, according to the sampling theorem, the lateral resolution of this configuration cannot exceed 5.16  μm. For this reason, the initial setup is still considered the best compromise between FOV, axial, and lateral resolution and will be considered more in-depth for the following analysis.

## 5 Discussion

The characterization of the non-modified setup revealed a very good performances of the microscope, which reached an isotropic resolution of 2.4  μm. We have shown that micrometer-sized structures such as axons and dendrites in thick samples can be resolved with our system without compromising the acquisition speed; acquiring three-dimensional images with an isotropic resolution of about 2  μm is done at a rate of up to $8.61\;{\mathrm{mm}}^{2}/s$. We therefore have an imaging system that not only allows three-dimensional imaging of large volumes at high isotropic resolution but also has a fast acquisition time due to its very wide FOV. This achievement can be attributed to the judicious combination of Bessel-Gauss beams, two-photon fluorescence and an amplified femtosecond pulse laser. The combination of our light sheet technology with optical clearing techniques promises to transform our ability to understand the brain circuits and therefore advance the understanding of neurological and psychiatric diseases that involve the remodeling of brain connections.

The use of Bessel-Gauss beams does not only have the advantage of resolution performance. Using these beams, the energy is also better distributed in the FOV, thus minimizing the risk of photobleaching or damage to the sample.

As we can see in Table 1, the characteristics of our system exceed those of the pre-existing light sheet systems. In this comparison, the lateral resolution is not used as a criterion as it depends entirely on the detection objective and the camera used. What really differentiates the systems is the light sheet generated in the sample as its extent determines the maximum FOV and its thickness determines the axial resolution of the system.

**Table 1:** Comparison of the performance of our system with other published technologies. The comparison is based on two factors, the light sheet extent, which is determined by the effective length of the beam used, and the light sheet thickness, which is usually equivalent to the axial resolution. Another commonly used metric is the lateral resolution; however, since it is usually only dependent on the detection objective and the camera, it is omitted in this table.

Published by	Light sheet extent (μm)	Light sheet thickness (μm)
Voie et al.	750	26.1
Huisken et al.	660	3 to 10
Dean et al.	216	0.39
Planchon et al.	80	0.3
Olarte et al.	246	2.8
Buytaert et al.^34^	7.7	2
Takanezawa et al.	600 to 1,000	2 to 3
Glaser et al.	29.1	2.91
**Large FOV**	1,312	2.4
**Ultra large FOV**	5,845	2.4

Most of the previous reported light sheet systems use Gaussian beam illumination, which only allows small light sheets of less than 500  μm to get a micrometric axial resolution (Table 1). For this reason, the only comparable system in terms of the two chosen criteria to our large FOV microscope is from Takanezawa et al.^26^ as it also uses Bessel-Gauss beams and two-photon excitation. They obtained a light sheet with a thickness similar to ours, but the extent of the sheet is much smaller. Their system also has a similar fast acquisition rate that depends on the exposure time, varying between 100 and 300 ms. However, since the energy pulse (350 nJ) of their laser is lower than ours (4  μJ), it is reasonable to assume that it would not be possible to extend their sheet dimensions to a few millimeters. Indeed, the nearly 6-mm sheet obtained with the ultra large FOV setup is made possible, among other things, by the high-power laser pulses that are required to create fluorescence over such a long distance.

Our ability to enlarge the light sheet to a size of nearly 6 mm significantly increases the acquisition rate, but more importantly, it avoids the stitching process, which is necessary to recreate large volumes from small FOV images. This procedure is often time consuming and computationally demanding, at the expense of the lateral resolution, which is most often limited by the camera used. Due to the large FOV on our system, there are no sCMOS cameras on the market presently that have a small enough pixel size and a high enough pixel count to achieve a better lateral resolution. However, with the growing interest in large volume light sheet microscopy, the next few years will surely bring new developments in camera performance, perhaps allowing our method to reach a lateral resolution higher than 2  μm, making this ultra large FOV system truly isotropic.

## 6 Conclusion

In this paper, we introduced a new two-photon light sheet microscope with features that surpass the performance of the existing systems. By taking advantage of an amplified femtosecond laser and an axicon lens, the system is able to acquire large 1.312  mm×1.312  mm FOV images with an ∼2  μm isotropic resolution and a fast acquisition rate up to $8.61\;{\mathrm{mm}}^{2}/s$. Additionally, by decoupling the diameter and the length of the Bessel-Gauss beam produced by the axicon, we modified the setup to extend the focal line and reach an unprecedented 5.845  mm×5.845  mm FOV. In this configuration, the acquisition speed was greatly increased to $34\;{\mathrm{mm}}^{2}/s$ and the optical sectioning remained as low as 2.4  μm. Both of the normal and modified systems were demonstrated by imaging the dopaminergic system and its axon projections in a whole cleared mouse brain. Now that several millimeter wide light sheets are possible, we look forward to future advances in CMOS cameras technology that will allow our system to achieve even higher lateral resolution.

## Code, Data, and Materials Availability

All the data and images used in this study have been deposited on our web site.^35^
